# Remarks on a Peculiar Appearance of the Tongue in Malarial Disease; Read before the Greensborough Medical Society

**Published:** 1851-08

**Authors:** Thomas C. Osborne

**Affiliations:** Erie, Ala.


Art. II.—*Remarks on a peculiar appearance of the Tongue in Malarial Diseases;* read before the Greensborough Medical Society. By Dr. Thomas C. Osborne, of Erie, Ala.

Fever has been a subject of paramount interest in all ages of medicine. It has been the theme of endless discussion and of numberless dissertations, but has lost none of the importance attached to it by its earliest investigators. Its cause continues to be a matter of speculation; its pathology is still far from being settled; and the mortality arising from it, though happily diminishing, remains very great. Under these circumstances any new fact contributed to our knowledge respecting its pathology, cure, or prevention, cannot fail to awaken interest. It is to direct the attention of physicians to certain phenomena in miasmatic diseases which I think I have observed, that these pages have been prepared.

The medical world has been divided between the *idiopathic* and *symptomatic* theories. A still greater diversity of views has prevailed in reference to the cause of fever, but I shall not stop to discuss them. The vegeto-animalcular hypothesis seems to me to have most to recommend it, and as it is well set forth by Dr. Drake in his able work on the diseases of the interior valley of North America, I trust I may be pardoned for quoting at some length his remarks on the subject.

On page 723 of that volume, he says: "The microscope has revealed the existence of a countless variety of organic forms, which surround and penetrate the bodies of larger animals and plants, whether living, or dead and decaying, inhabit all waters, salt and fresh, and swarm in the atmosphere; buoyed up and moving by their own organs, or sustained by their levity, and wafted about by currents of air.....The power of reproduction possessed by these microscopic creatures, is still more wonderful than their minuteness. It exceeds, indefinitely, all examples presented by the visible organic kingdom; where, however, we see the government of the same law, for, in both plants and animals, the small multiply more rapidly than the large.....Among the visible plants and animals, there are species that form no poison, and others which secrete that, which applied to, or inserted in our bodies, produces a deleterious effect, which is generally of a definite kind. Thus, the venom of the rattle snake produces a disease of a definite form; cantharides another; certain fish are poisonous when eaten; wasps and bees instil a venom; and the smallest visible gnat, as that which inhabits the forests of the middle latitudes, and that which is known under the name of the sand fly on the shores of the Gulf of Mexico, inflames the skin; while the juice of the stramonium, the exhalations of the rhus toxicodendron, and the fungus which grows beneath its shade, excite peculiar diseases. It seems justifiable to ascribe, by analogy, to microscopic animals and plants, the same diversity of properties which we find in larger beings, differing from them, as we may presume, in nothing but size and complexity of organization."

Again, on page 726, he says: "Microscopic observation and analogy render it probable, that in the invisible, as well as the visible province of the organic kingdom, there are distinct species, which constitute, by their union, natural families or orders. We know that in each natural assemblage of the larger plants and animals, the species resemble each other in many internal qualities, as well as in their forms. Thus, an astringent principle pervades the various kinds of oak; a resinous principle the linear evergreens; an aromatic oil the peppermint; and other didynamous herbs; a poisonous principle, the different species of rhus; and that a narcotic principle pervades a large assemblage of plants. Now, may it not be, that two distinct species of the same natural order of microscopic beings, may produce autumnal fever? May not one be the cause of intermittents—the other of remittents? may not both act on the system at the same time; and may we not thus explain diversities, which are inexplicable on the malarial hypothesis. Every practical physician knows, that while the juice of a variety of plants will produce the pathological condition called narcotism, the symptoms of that state, when induced by the different agents, differ as widely from each other, as the symptoms of the different forms of autumnal fever."

But whatever be the poison generating fever—whether malaria, animalcules, or cryptogamous plants--if I am not mistaken in my observations I have remarked an appearance of the tongue, which reveals the presence of the poison in the system in time to enable us to remove it before it has exerted any seriously deleterious influence.

My experience is, that this earliest evidence of the impress of the poison is uniformly present, and that we may ward off the attack by timely attention to it.

For several years past, my attention has been attracted to this peculiar condition of the tongue, which from its invariable position and presence in the diseases and predispositions generated by miasma, may be appropriately denominated the *malarial margin.* As the name imports, it is an essential departure from the normal aspect of the edge, constituting a distinct lateral boundary of the tongue, occupying more or less surface according to the charge of infection in the system. Ordinarily, the color amounts only to a very faint bluish tinge, which is liable to be lost, or merged in the various tints imparted to the tongue by various diseases. The most fixed condition of this symptom is an appearance of indentation or crimpling transversely, which is apparently confined to the subjacent tissue, while the superficial tegument is moist, smooth, and transparent. In word, it seems to be a continuation or encroachment of the inferior surface upon the superior and lateral borders of the tongue, greater as we approach the root of that organ.

My opportunities for noting this appearance of the tongue have been ample during several years, among a considerable population composed of the white, black, and mixed races permanently residing upon the banks of the Warrior river, (in sandy as well as the prairie regions) and in many individuals from other and different sections of this and the surrounding states; and I can truly say that in no instance has it ever failed to inform me correctly of the agency which the remote cause of fever had begun to exert in each individual case. After being once recognized it is not easily mistaken, and from pointing it out so frequently to non-professional persons of my acquaintance, many of them have become as thoroughly convinced of its significance as myself, and call it, very properly the

"fever tongue." I have frequently, upon seeing a well defined instance of the malarial margin, predicted fever within a month, and although the individual would be disposed to smile incredulously, and boast of excellent health, the result has generally created in their minds and bodies a different sensation.

The fidelity of this symptom to the source of its origin is under all circumstances fully equal to the importance which I have attached to it. In a considerable number of cases of the different forms of dropsy, neuralgia, and inflammation, it has alone enabled me to reach a correct diagnosis, when every other symptom seemed to deny the agency of malaria in the case. Wherever seen, I have invariably assumed that there existed a tendency to intermittent disease, and upon watching the progress of the case have as invariably detected this condition. In numerous cases of pulmonary inflammation, where the fever seemed continued, the cough unabated, the oppressive respiration persistent, and the pulse unvarying in its activity, the physical signs have designated with great accuracy the periods of repose and excitement in the course of the disease. In the gastric and intestinal phlogoses, again, whether as causes or consequences of fever, if this peculiar impression is seen upon the tongue, no apprehension need be felt in the administration of quinine, however malignant the case may appear. Here also intermission, varying in degree according to the severity of the disease, is always present, and generally speaking, the shorter the period of repose the greater the quantity of medicine required, and *vice versa*. I have not hesitated, in many cases, where the disease was marching rapidly to a fatal termination, to prescribe one, two, and even three drachms of sulphate of quinine at a dose, to be repeated according to the urgency of the indications. Gastro-enteritis as a complication in the autumnal fevers of our section is by no means uncommon. On the contrary, in the limits of my practice it exists in a majority of cases. At the present time there prevails on both sides of the Warrior an epidemic diarrhea, as symptomatic of mucous irritation, which in a number of instances resists all the usual remedies given in such cases, but yields readily to repeated doses of quinine, and the daily use of the cold dash. The most active of these cases present unmistakeable evidences of gastro-intestinal inflammation of a high grade, but in no instance have I failed on a close examination to recognize a quotidian or tertian form of intermittent, and in no instance has the malarial margin been absent from the tongue.

My tabular report of the diseases of the last month exhibits diarrhea as the prevailing type of disease, and by reference to that paper it will be seen that there have occured to me twenty-five cases, in individuals varying in age from three months to sixty years; as respects the treatment, the whole number may be divided as follows, viz:—

Of those between the ages of three months and ten years, six required quinine and the cold bath, while five yielded to the administration of opiates and astringents; and of those over ten years, twelve demanded quinine and the cold dash, while only two yielded to opiates and astringents.

Some of these cases were of an alarming character, and my conviction is that their favorable termination is due to the use of quinine which I was at first led to administer by the malarial tongue. During the last four years our country has been visited by an unquestionable form of typhoid fever, and in not one of the cases of this disease which I have seen has the tongue exhibited the malarial border, but has appeared as if scalded in patches. But the semblance of typhoid fever is assumed by many cases of the autumnal disease, in which, but for this symptom, much apprehension might be entertained and much valuable time lost to the physician in the treatment, for it is well known, that quinine is actually injurious in all stages of the typhoid fever, while in those cases merely wearing its livery, that remedy is not only useful but essentially necessary to the cure of the disease.

If the malarial margin were useful only in unmasking this form of disease, its value would be inestimable; but when it points out with equal certainty all other varieties of disease in which miasma exerts a predisposing agency, it becomes a phenomenon of the greatest importance.

It is not easy to exaggerate the value of a sign by which we detect the operation of the poison in its inception, and are conducted to the true method of suppressing it. I subjoin a few cases illustrative of the utility of this diagnostic feature :—

Case 1. *Malarial Dyspepsia.*—A planter who has resided at his present abode for seven or eight years, had generally enjoyed tolerable health up to September, 1849. He was then attacked with a severe autumnal fever of the typhoid type, from whieh he gradually recovered in about four months. In the following spring he began to experience dyspeptic symptoms, which were usually accompanied by a diversity of complaints in the nervous and tegumentary systems, whenever he indulged too freely at table or in diet that disagreed with his stomach. Sometimes there was distressing pain in the head and back, attended with weakness and tremors of the extremities; at others lumbago, with transitory arthritic pain; or the affliction would under favorable circumstances invade the mucous or cutaneous surfaces and excite in the one temporary painful eruptions; or an irritation would occur in the mucous system and give rise to catarrhs, asthma, or diarrhea, etc. These attacks, when once established by inattention to dietetic regulations, would always observe, in their progress, a regular series of exacerbations and remissions, either quotidian, tertian, or quartan in type, during several periods; each growing lighter, unless arrested by the use of quinine or peruvian bark, which constitute the principal part of the treatment in every instance. They continue still to recur occasionally, but are weaker, more transient in duration, and observe longer intervals of repose than formerly. Since the attack of fever, his tongue has retained the malarial margin persistently, while its configuration and other appearances have undergone a great variety of alterations. There is also one other symptom in this case which I have not witnessed elsewhere. For several hours, or even days before the attacks of either of these forms of sickness, and during their course, a patch or patches of bright red discoloration, in various sized spots—from a mere stigma, to the dimensions of a dime—of a circular form, with perfect intervals of natural surface, occur on the palmar side of the hands, being most distinct on the fleshy mass near the thumb. He learned early to appreciate this rash as a faithful premonition, and, upon their appearance uses the means to ward off the violence of the suffering which is sure to follow.

His residence is on an elavated south bank of a lagging stream, which courses meanderingly a little south of west, through open fields, to the Warrior river, distant some three or four miles. With the winter and spring freshets this small creek spreads its waters freely over a considerable surface of adjacent low lands on both sides, and the layers of decomposing sediment thus deposited enjoy very favorable exposure to summer heat. Immediately on the east, and adjacent to the dwelling, commences a chain of hills which are heavily timbered; and on the north, hard by, there is a body of low alluvion comprising several hundred acres, densely shaded, but having a great surplus of dead and decaying vegetation upon it.

Thus, he may be said to live at the head of navigation of the remote cause of fever, and as the prevailing winds of summer and autumn are from south and west, much sickness of this character might be reasonably expected in his large family; and facts go to verify the supposition.

CASE II.—*Malarial Dropsy.* Mrs. S. was attacked with anasarca on the first of January, 1851, during pregnancy, and at the eighth month gave birth to a puny child, early in March.

The dropsy had before been strictly confined to the cellular tissue, but now increased rapidly and in a short time filled the thoracic and abdominal cavities to a very painful degree of distension. No relief could be obtained, nor sleep procured, from the oppressive dyspnœa except in a sitting posture. In this condition she remained until the twenty-ninth of May.

Upon an examination of the tongue I found a wide malarial border, and by pertinent inquiry ascertained that she was suffering hebdomadal paroxysms, this being the day for one of the periods. The surface was jaundiced, the flesh wasted, the respiration short, heaving and painful; pulse faint, frequent and thready; the temper morose and fretful, bowels active from mercurials that had been repeatedly administered, and the urine scanty and high colored. Of course she had not been able to nurse the babe at the breast, and it was shadowy and sinking. Prescription:—

℞ Pulv. citrat. ferri,

Sulph. quin. ää grs. v, in pills, three times daily for four days; then every two hours for two days.

Pulv. digitalis j gr. every six hours.

Decoction of uva ursi and buchu leaves ii oz., every night.

Tepid bath daily.

Thursday, June 5th. Bowels still brisk; kidneys aroused to powerful action, the urine being limpid and passing off hourly in great quantities; pulse better developed and reduced in frequency; the breathing more easy and natural; able to rest quietly and sleep upon the bed. Continue the medicines.

Sunday, 8th. Peculiar effects of digitalis manifested strongly, commencing on yesterday; the whole system prostrated under its influence; pulse irregular, full and intermittent; respiration natural; urine discharging freely; intense pains in the stomach and bowels, and bilious vomiting. *Dropsy disappeared.* Discontinue medicines, and give brandy and wine whey; blister to epigastrium.

Wednesday, 11th. Effects of digitalis continue, but gradually dying out; pains and vomiting arrested; urine less copious, and high colored again; skin cleared up.

Auscultation revealed, from the first day of digitalis impression, a prolonged bellows murmur, during first third of the heart's action, completely masking the dull sound, while a tremulous agitation of the chest was communicated to the hand with every impulse or shock of the heart. As the effect of the medicine approached its maximum, the abnormal sounds increased in intensity, and the murmur resembled exactly the thrust of a coarse saw.

To-day the sounds are less rough, or in other words as the effect of the medicine diminishes the abnormal sounds disappear, and are replaced by an approach to the natural order of sounds in the heart's action.

Stimulants gradually discontinued; recommence sulph. quinine and citrate of iron; paint surface of abdomen and thighs with tinct. iodine daily; light diet.

Friday, 13th. Convalescent. I should have stated that during the paroxysms of pain, chloroform, by inhalation, was given repeatedly with the most pleasant results. She keeps it now constantly at hand. The residence of this lady is on the west side of the Warrior, and immediately on the border of a sluggish slough, the waters of which are dammed up by every rise of the river, and during the hot seasons give rise to offensive effluvia all along its course.

CASE III.—*Malarial asthenia of the uterus.* Mrs. M., aged forty-five years, was taken with menorrhagia in the fall of 1846, at a period of the catamenial flow, which continued rather passively until she was reduced to the verge of the grave. The next year it returned in the summer; and the next, again, in the middle ot spring, and in each visitation the duration was prolonged to the approach of cold weather. In 1849, it returned early in the spring, and was of so threatening a character that her physicians advised a removal to some of the established watering-places; and after a month or two stay, under the use of chalyebate drinks, and healthy atmosphere, her health rapidly returned, and the hemorrhage ceased. She returned home in September, and was in a short time suffering again from the disease. In November I saw her for the first time. She was now wasted in flesh, and her complexion entirely replaced by sallow paleness; the pulse was feeble and frequent; the muscular energies prostrated; the digestive powers exhausted; and the stomach so weak that very few things taken upon it would remain. The tongue was pale, and had a broad malarial margin, and upon inquiry I found that she had evening exacerbations of fever. This state of things she assured me had existed all the time, save in the interval passed at the springs. The several physicians who had been in attendance pronounced it an approach to the critical period, and when I ventured to assure her that under appropriate treatment, health might be restored in a few weeks, she manifested the utmost incredulity. But finally, she became convinced that to wait for the critical period to pass would be to wait for certain death, and consented to be put upon a curative treatment at once.

Sulphate of quinine was given in six gr. doses every two hours every morning—a solution of citrate of iron was ordered as a constant drink—opium and acetate of lead, which had been previously rejected time after time by the stomach, was now given with the very best effects, and in two weeks the amendment was so great that I discontinued my attendance. Every few months since, during the hot season, the hemorrhage is disposed to return, but a few doses of quinine quickly restores tone to the system and especially to the uterus. This lady lives immediately upon the east bank of the river.

Cases IV and V.—*Malarial palpitation of the heart.* Mrs. M., is of the leuco-phlegmatic temperament; is about thirty years of age; has been living for several years inside a large clearing, immediately upon the border of a switch cane marsh; and is visited every year by one or more spells of fever. For several months previous to each of these visitations her health declines, and she is troubled with fits of palpitation without any physical sign of disease of the heart. The paroxysms return with great regularity every 7th, 14th or 21st day, until she gets well from the attack of fever, when they disappear. I have been the family physician for more than two years, and have always found the malarial margin upon the tongue, and have in every instance succeeded in arresting the fits of palpitation by the use of quinine.

Mr. S., aged thirty years, came to live upon the borders of the river, as a planter, in February last; having spent much of his time, during the summer season at mountain watering-places, and his health might be said to be good up to the period of his removal. I saw him just before he moved, and requested a sight of his tongue; I found it in a healthy condition. In March just one month after, he was attacked with a severe fit of palpitation, which gradually passed off in perspiration. The third day subsequently he had, at the same hour, another attack and sent for me. The malarial border was now broad upon the tongue, and quinine arrested the disease.

Does not this case prove the generation and action of the specific poison during the winter and spring months?

Case VI.—*Malarial Dysentery.* A family of previously healthy persons, eighteen in number, living upon the margin of a foul swamp, were nearly all attacked successively with acute dysentery in June 1850, and two of the stoutest of the white members died of that complaint. After these deaths I was called in, and finding the malarial margin well developed, although the tongue was fiery red and encrusted as in all such cases, I put them upon the use of quinine and all recovered in a short time.

Case VII.—*Malarial Pneumonia.* A negro man, 22 years of age, living in one of our proverbially sickly places, was attacked violently with inflammation of the lungs on the 15th of March, 1851. I did not see him until next day; he was then easier, but the physical signs indicated a rapid progress in the disease, and it was with great difficulty that he could breathe. The tongue, although red and dry, had the malarial border upon it. He was bled freely, and cupped extensively over the seat of the inflammation, and, after that, blistered in the same region. Next day he grew worse—the next, better. Quinine, in fifteen grain doses, being now given, he rapidly recovered. Cases of this character were numerous last spring, but as one is enough to exhibit their nature and the course pursued in each, I will refrain from inserting in this place any others.

Case VIII.—*Malarial Cerebro-Meningitis.* A bright mulatto girl, aged 15 years, a field hand, had had chills and fever repeatedly during the autumn and winter of 1850, and on the 31st of January last, in the midst of a severe spell of cold inclement weather, the disease returned, attended with symptoms of cold, and acute headache. I saw her the next day, and found the fever passing off in a free perspiration, all the symptoms being greatly relieved. The tongue was thickened, pointed, and had the malarial border upon it; and as a mercurial had been given, I merely ordered rest in the horizontal posture, and quinine as in intermittent fever.

February 2d. Fever returned, without a chill, early this morning; pulse 150, small and feeble; conjunctiva and vagina discharging freely; mind confused; vigilant; head very painful; acutely sensible to light and sound; pupils strongly contracted; paralysis of the right arm and left leg, the surface of these parts being morbidly sensitive to the touch, or even to a current of air; bowels acting, the evacuations consisting of tar-like bilious matter; urine scant, involuntary, and strong in odor; restlessness and constant muttering.

Prescription—Venesection; cups to temples and nape; blisters to extremities.

℞. Ipecac. pulv., grs. iii, every 2 hours.

3d. Intermission perfect; paralysis disappeared; quite cheerful; hungry; and expresses desire to leave the bed. Sulphate of quinine in x grain doses every three hours during the day.

4th. Fever rose early, and runs very high; paralysis returned; all the symptoms aggravated. On inquiry I ascertained that quinine had been given every three hours, but the nurse thought a solution of that medicine in whisky was the best way it could be given, and that a tablespoonful of this preparation would weigh about ten grains, and accordingly administered it in that way.

Death on the 5th, in convulsions.

*Necroscopy, twenty-two hours after death.*—Dura mater dull and lead-like in color, adhered tenaciously to the subjacent tissues all along the superior edges of the hemispheres, from the anterior to the posterior of the cerebrum. In dissecting these adhesions four groups of tubercles lay imbedded in the adventitious membrane, each group having about fifty of these bodies, which, in size and color, resembled the large white mustard seed, and were as firm as fibro-cartilage. Near each group, especially on the anterior lobes, there were several dots of milky-looking pus. The arachnoid and pia mater displayed everywhere a diffused scarlet blush, which was intersected in all directions by a vascular net-work of a crimson color. Two rays of recent lymph on each side of the anterior lobes, one and a half inches in length and two lines in width, shot out from the membranous attachment on the edges of the hemispheres, and were of a dull straw color, and confined to the sub-arachnoid cellular tissue. The cerebrum, cerebellum, annular protuberance, and medulla oblongata exhibited intense capillary engorgement, with red indellible punctuations in every imaginable direction, but no other lesions of any kind. Brain only examined.

Note.—At the close of the reading of this paper the members of the Society manifested very considerable interest in the peculiar appearance of the tongue spoken of, and on examination the writer was able to exhibit three specimens of it among the gentlemen present. The members then declared themselves satisfied of its existence.

June 23, 1851.

