# Erratum

**Published:** 1851-08

**Authors:** 


Erratum.—In the paper of Dr. Osborne, for "*drachms,*" read *half* drachms of sulphate of quinine.

